# Quantitative analysis of the impacts of terrestrial environmental factors on precipitation variation over the Beibu Gulf Economic Zone in Coastal Southwest China

**DOI:** 10.1038/srep44412

**Published:** 2017-03-15

**Authors:** Yinjun Zhao, Qiyu Deng, Qing Lin, Chunting Cai

**Affiliations:** 1 Key Laboratory of Environment Change and Resources Use in Beibu Gulf, Ministry of Education, Guangxi Teachers Education University, 175 Mingxiu east st, Nanning 530001, China; 2 School of Geography and Planning, Guangxi Teachers Education University, 175 Mingxiu east st, Nanning 530001, China

## Abstract

Taking the Guangxi Beibu Gulf Economic Zone as the study area, this paper utilizes the geographical detector model to quantify the feedback effects from the terrestrial environment on precipitation variation from 1985 to 2010 with a comprehensive consideration of natural factors (forest coverage rate, vegetation type, terrain, terrestrial ecosystem types, land use and land cover change) and social factors (population density, farmland rate, GDP and urbanization rate). First, we found that the precipitation trend rate in the Beibu Gulf Economic Zone is between −47 and 96 mm/10a. Second, forest coverage rate change (FCRC), urbanization rate change (URC), GDP change (GDPC) and population density change (PDC) have a larger contribution to precipitation change through land-surface feedback, which makes them the leading factors. Third, the human element is found to primarily account for the precipitation changes in this region, as humans are the active media linking and enhancing these impact factors. Finally, it can be concluded that the interaction of impact factor pairs has a significant effect compared to the corresponding single factor on precipitation changes. The geographical detector model offers an analytical framework to reveal the terrestrial factors affecting the precipitation change, which gives direction for future work on regional climate modeling and analyses.

Climate change has been a topic of worldwide concern in recent years. Precipitation is the most active parameter of all the meteorological elements. A large number of studies show that precipitation exhibits change in many areas^1^,^2^. Precipitation change caused by the anomalous change of atmospheric circulation is a very complicated phenomenon, which is primarily the result of internal adjustment of the atmosphere itself. However, in terms of the regional scale, the terrestrial environment will respond to precipitation change through land-atmosphere interactions. It should be noted that, to some extent, terrestrial environment impact is comparable to atmospheric circulation and solar radiation.

The terrestrial environment primarily includes natural (vegetation coverage, vegetation type, terrain and terrestrial ecosystem type) and social (human activities) factors. Vegetation coverage impacts the climate through its effect on surface albedo^3^, etc. Compared to surrounding areas, the ground vegetation properties in the region, such as surface albedo, roughness and soil humidity, have a large variation that would influence local thermal and moisture conditions. This, in turn, changes local precipitation through atmospheric circulation on a small to medium scale. Previous research showed that precipitation can increase^4^,^5^,^6^ with the growth of forest coverage. Different vegetation types also have distinct impacts on the surrounding climate^7^. For example, a coniferous broad-leaved forest and its analogues have a greater impact than other vegetation types on the change of the average annual precipitation trend^8^. The terrain will affect the entire atmosphere precipitation system^9^. The slope, altitude, latitude and other similar factors will directly affect the precipitation by changing the regional atmospheric circulation^10^,^11^. The terrestrial ecosystem influences the concentration of greenhouse gases and aerosols in the atmosphere, thus affecting climate change through the energy balance between the ground and the atmosphere, the interaction of water vapor exchange and the biogeochemical cycle^12^. At the same time, the ecosystem would respond to the climate change^13^,^14^, namely, both climate interactions have mutual effects. Human activities, such as agricultural irrigation^15^,^16^,^17^,^18^,^19^,^20^,^21^ and urbanization^22^,^23^, directly or indirectly exert some impact on the precipitation distribution by changing regional underlying surface hydrothermal conditions to affect atmospheric circulation. For example, urbanization in Guangzhou accounts for 44.7% of the significant precipitation growth since 1991^24^; irrigation increases precipitation while decreasing the daily average and maximum air temperatures^25^,^26^.

Most of the aforementioned researches have analyzed the change in the characteristics, temporal-spatial trends and impact factors of precipitation (climate) in a certain area^27^,^28^,^29^,^30^,^31^,^32^,^33^; however, these researches are lacking quantitative analysis on the combined effects of multiple important factors under a unified analysis framework. Based on the spatial variation theory, the geographical detector model^34^ was used initially for evaluating the relationship between health and suspicious pathogenic factors. It can measure the spatial consistency and statistical significance between health risk and geographical elements and determine the effectiveness of the spatial correlation without many assumptions. It also effectively overcomes the limitations of processing category variables that exist in the traditional statistical analysis method^35^. Thus, the application of the geographical detector model has been gradually extended to other areas, such as resources and the environment^35^,^36^,^37^,^38^,^39^,^40^,^41^, for quantitative analysis of the mutual relationship between the factor and result variables^42^,^43^. It should be mentioned that the geographical detector model has never been utilized to provide an analysis framework in order to study precipitation change.

Therefore, this paper attempts to answer the following questions: First, what is the major determinant affecting precipitation change? Second, does each factor affect precipitation change independently or interactively? Third, what is the relative importance of these affecting factors? The Guangxi Beibu Gulf Economic Zone was selected as the case study in this research. The Guangxi Beibu Gulf Economic Zone is located in the southwest China coast and consists of the administrative regions of Nanning, Beihai, Qinzhou, Fangchenggang, Yulin, and Chongzuo city (Fig. 1). The land area covers 425,000 km^2^. The Beibu Gulf Economic Zone is located south of the Tropic of Cancer and is a subtropical maritime monsoon climate zone with transitional characteristics from tropical to subtropical. The annual average temperature ranges between 21.5 °C and 23.4 °C, while the average daily temperature stabilizes above 10 °C. The multi-year average precipitation ranges between 1251.27 mm and 2717.87 mm. The Dongxing-Qinzhou region on the southeast of Shiwan Dashan Mountain is one of the three rainy districts in Guangxi. The precipitation amount during flood season, generally from April to September, accounts for 80% of the annual precipitation, with peak precipitation occurring in July and August.

## Results and Interpretation

### Precipitation change in the Beibu Gulf Economic Zone

The precipitation trend of each meteorological observation (88 total meteorological observatories in Guangxi Province) was calculated from annual precipitation for the period of 1985~2010 using Equation (1). Linear trends indicate that 76% of the meteorological stations show a positive trend in annual precipitation during 1985–2010, and notably, five of them are statistically significant at the 90% confidence level (see Supplementary Table S1, Fig. S1). The other 24% of the meteorological stations show a negative trend (see Supplementary Table S1). A station-by-station analysis was performed and mapped using ArcGIS 10.1 with the Empirical Bayesian Kriging interpolation method in order to explore spatial patterns of precipitation changes in Guangxi Province. Then, the precipitation changes in the Guangxi Beibu Gulf Economic Zone were clipped and are shown in Fig. 2. This might be better than using direct interpolation of fewer meteorological observatories from the Guangxi Beibu Gulf Economic Zone, especially in the border area, because of the regional characteristics of precipitation.

Figure 2 shows that the precipitation trend rate of the Beibu Gulf Economic Zone is between −47 and 96 mm/10a. The character of the spatial precipitation change is primarily in the northwest-southeast direction. The low value zones are located in the southwest Beibu Gulf Economic Zone, while the high values are located in the northeast. The figure also shows that the precipitation changes in the middle zone are relatively lower than those in the neighboring south and north areas. A relative increasing precipitation trend from south to north is observed as a whole. We also found two zero lines of precipitation change in the middle and southwest zone of the Beibu Gulf Economic Zone.

According to Li & Su’s research, the Mann-Kendall mutability test found that precipitation in Guangxi Province had sudden changes in the years of 1984 and 1994. Specifically, from 1984 to 1994, Guangxi Province had less rain, while beginning in 1994, Guangxi Province entered into a relatively pluvial period^44^. Existing research also shows that there was a positive trend center of precipitation in northwest Guangxi. Therefore, in general, the linear trend is increasing.

### The feedback of terrestrial environmental factors to precipitation change

#### The leading factors of precipitation change

The factor detector ranked the terrestrial environment layers by their influences (P_D,H_ values) on precipitation change in the following order for the study area (Table 1):

FCRC (50.3%) > URC (47.3%) > GDPC (43.5%) > FRC (35.2%) > PDC (27.4%) > VT (10.0%) > DEM (7.3%) > GT (2.4%) > GRD (0.8%) > ASP (0.4%) > LUCC (0.3%) > TET (0.1%).

Among the terrestrial environmental factors, the P_D,H_ value of FCRC is the maximum. Obviously, there is a large break in sorted P_D,H_ values between PDC and VT. The P_D,H_ values of FCRC, URC, GDPC, FRC and PDC are in a group with high values and small differences, while the rest of the factors belong to another group with lower values. In a general sense, if the P_D,H_ value of a factor is larger than 0.2(20%), then the factor can be regarded as a leading factor^39^ that strongly explains the spatial pattern. Therefore, FCRC, URC, GDPC, FRC and PDC are potential leading factors that may exert the largest impact on precipitation change (spatial pattern) in this study area. In contrast, the values of VT, DEM, GT, GRD, ASP, LUCC and TET are comparatively small at less than 0.1(10%), which likely reflects their smaller contributions to the precipitation trend (spatial pattern).

The ecological detector (Table 2) shows the differences of the P_D,H_ values. Among the five potential leading factors (FCRC, URC, GDPC, FRC and PDC), approximately 80% of them (FCRC, URC, GDPC and PDC) are not statistically significant with each other, whereas statistically significant differences between FRC and other potential leading factors (URC and GDPC) were found. That is, URC and GDPC have a larger significant effect on the precipitation change than FRC. With the factor detector and the ecological detector, we concluded that FCRC, URC, GDPC and PDC are leading factors, and FRC was eliminated from the potential leading factors. Therefore, FCRC, URC, GDPC and PDC have the largest contribution to the precipitation change, whereas the remaining factors have a relatively weak influence.

In view of the above considerations, we also found that the power of social factors is much larger than that of natural factors in changing precipitation in the study area. This can be seen from the leading factors (FCRC, URC, GDPC and PDC) and whole sorting of the P_D,H_ values, which means that people are likely to be a very powerful factor in changing the terrestrial environment to influence local precipitation on a regional scale.

On the surface, FCRC is the first leading natural factor. However, the increase of forest coverage rate change in the study area is predominantly due to ecological construction for tourism and sustainable development in recent years. The forest coverage rate change of this study area is higher than the rate of other places in China and occurs in a sustainable growth manner. The Shiwan Dashan National Forest Park and the Daming Mountain National Natural Reserve are located in this research area. Some research has already highlighted that the significant role of precipitation may increase or decrease alongside afforestation^4^,^5^,^6^ or deforestation.

The Guangxi Beibu Gulf Economic Zone is the first international regional economic cooperation zone in China. According to the statistics, the population of this region was 15.81 million in 1985 and rose by 43 percent to 22.68 million in 2010. The GDP of this region increased from 8.3 billion Yuan (RMB) to 412.1 trillion Yuan (RMB) between 1985 and 2010, which is a huge growth of 49.37 times the initial GDP. With the sustainable growth of population and the increasing development of the economy, the GDP, the population density, urbanization, construction activities, energy consumption and greenhouse gas emissions have experienced a relatively rapid growth in the Beibu Gulf Economic Zone, and among them, the GDP growth rate is the largest.

The urbanization levels of both Nanning City and Beihai City are over 55%. In contrast, Guangxi has a relatively extensive development model for their economy, with an energy consumption per unit of GDP of 1.036 tons of standard coal per ten thousand Yuan (2010), which is 1.28 times the national average of 0.81 tons of standard coal per ten thousand Yuan. A large amount of energy consumption emits a large amount of greenhouse gas, such as carbon dioxide, which is the main source^45^ of carbon emissions. This greatly influences the climate of this region and possibly the climate on a larger regional scale. Urbanization is a comprehensive process, which will influence a city’s precipitation, temperature, humidity, visibility and wind, forming a special local meteorological environment and causing material climate changes. The GDP may actually be viewed as a comprehensive result of many human activities. The increase of population density leads to an increase of artificial thermal discharge, directly influencing the change of surficial sensible heat flux, which will influence precipitation significantly^46^. In addition, approximately 45% of China’s farmland is irrigated farmland^47^, whereas Guangxi Province has a higher percentage. The P_D,H_ value of FRC on precipitation change is 35.3% (much higher than 20%). This occurs mainly because the heavy irrigation of farmland affects the distribution of surface net radiation between latent heat flux and sensible heat flux change (latent heat flux increases, but sensible heat flux decreases), and farm irrigation has a cooling effect on the earth’s surface; at the same time, the increase of soil humidity enhances transpiration and further increases the moisture content in the atmosphere and the unstable energy of latent heat, leading to an increase of convective precipitation^48^ and producing a marked effect on the region’s precipitation. This finding is supported by other cases. Irrigation over the Ogallala Aquifer in the central United States increased dramatically over the 20th century and has enhanced regional precipitation^49^. The precipitation increase in the Texas Panhandle from 1952 to 1980 was obviously due to the increase in the irrigation area^25^. On the other hand, the amount of precipitation in central and southern India decreased due to a lower surface temperature over the irrigated areas of India in July^26^.

The Beibu Gulf Economic Zone is a relatively small area, which is on a small scale compared to the majority of research on precipitation. In the region, the DEM, geomorphic type, slope aspect, gradient, ecosystem and vegetation form are similar or experience less change, so they probably have a weak effect on precipitation change.

#### The effect of the interaction of terrestrial environmental factors on precipitation change

The interaction detector was used to check whether or not two factors work independently. The joint impacts of two factors measured by the P_D,H_ values are shown in Table 3 and Table S2 and can be compared with their separate impacts.

It must be noted from Table S2 that the P_D,H_ values of 22 interactive pairs are greater than that of the primary leading factor (FCRC). The max P_D,H_ value comes from interaction of FRC with GDPC (FRC ∩ GDPC = 84.4%). Specifically, all the interactive effects between FCRC and the rest of the factors (FCRC ∩ GDPC = 83.1%, FCRC ∩ FRC = 76.5%, FCRC ∩ URC = 74.1%, FCRC ∩ PDC = 75.0%, FCRC ∩ DEM = 57.3%, FCRC ∩ GRD = 53.6%, FCRC ∩ ASP = 50.9%, FCRC ∩ TET = 51.0%, FCRC ∩ GT = 54.8%, FCRC ∩ VT = 59.8%, FCRC ∩ LUCC = 51.0%) are stronger than the effect of the single FCRC (50.3%, the strongest effect on precipitation changes). We found that FCRC interacting with any other factors is always enhanced. Similarly, all the interaction effects between URC and the rest of the factors are higher than the single URC (47.3%) effect. Even of those factors with the lowest P_D,H_ values, interactions between them enhance their separate effects on precipitation changes. In general, all interactive pairs of impact factors showed enhanced results compared to the corresponding single factor, and among them, 45 interactive pairs have P_D,H_ values larger than 0.2 (20%).

The top P_D,H_ values of interactive pairs are FRC ∩ GDPC = 84.4%, FCRC ∩ GDPC = 83.1%, FRC ∩ URC = 77.0%, FCRC ∩ FRC = 76.5%, FCRC ∩ PDC = 75.0%, FCRC ∩ URC = 74.1%, URC ∩ GDPC = 72.3%, URC ∩ PDC = 71.0%, FRC ∩ PDC = 70.1% and GDPC ∩ PDC = 70.0%, and all of them are larger than 70%. We thought that FCRC is also a social factor because FCRC is mainly due to human ecological construction. Therefore, these factors are all social factors, and it clearly implies that humans are the most important aspect in changing precipitation (similar to the analysis of leading factors) in this region via economic activities such as urban construction, afforestation, changing and developing hillside fields, irrigation and plantation. Under the high pressure of growing population and development, humans are the best medium compared to other natural factors to change and affect the spatial distribution of other factors according to their purposes, and with the development of science and technology, this situation is amplified. For example, large-scale afforestation in the northern mid-latitudes warms the Northern Hemisphere and alters global circulation patterns to redistribute the anomalous energy absorbed in the northern hemisphere, which results in a precipitation decrease over parts of the Amazon basin and an increase over the Sahel and Sahara regions in Africa^50^.

In addition to the above mentioned, we also noted that interactions between social factors and natural factors have two types: nonlinear enhancement and bienhancement (Table 3). Each type indicates that the factors bienhance or nonlinearly enhance each other. As shown in Table 3, the interactions between social factors and natural factors have predominantly strong, nonlinear synergies. For example, the interactions of PDC and DEM (PD ∩ DEM = 40.3% > 34.7% = PDC (27.4%) + DEM (7.3%)) are larger than the P_D,H_ value sum of PDC and DEM; therefore, the interaction between PDC and DEM has a larger impact on precipitation changes. This is likely due to the city and farmland expansion toward a relatively bad condition of DEM that changes the underlying surface conditions. It also indicates that social factors and natural factors have synergies and can enhance each other’s effect on precipitation change.

In conclusion, social factors have a larger impact on the precipitation change compared to natural factors. Partial natural factors have a relatively small impact on precipitation change but show a strong synergy with the interaction of other factors. The feedback of terrestrial environmental factors on precipitation change mainly arises from interactions of impact factors and interactive pairs of impact factors, which have a larger influence on precipitation change than the single factor does through the feedback. Interactions between factors play an important role in the precipitation change in this region.

#### Regional analysis of the leading impact range (type) of leading factors on precipitation change

The risk detector shows that the average precipitation change in the different FCRC zones (from I to VI) are −0.66 mm/10a, −9.98 mm/10a, 53.18 mm/10a, 6.88 mm/10a, 23.48 mm/10a and 11.66 mm/10a, respectively, and they are significantly different. It also implies that precipitation will increase or decrease with the increase or decrease of forest coverage. However, higher precipitation change is not consistent with a larger FCRC zone, and precipitation change fluctuates with FCRC values. A similar analysis of other terrestrial environmental factors can be conducted using the risk detector. The small and continued growth of annual urbanization rates will lead to a large increase in annual precipitation. The main impact ranges of FCRC, URC, GDPC and PDC tend to be located at the relatively low-middle value zones. We selected the largest types (ranges) of each leading factor as the main impact types (ranges) by sorting the average precipitation change. The main impact types (range) are tabulated in Table 4 and mapped in Fig. 3.

From Table 4, we can see that the main impact types (ranges) of FCRC, URC, GDPC and PDC are 0.7411~4.7979%/10a, −7.7920~2.5006%/10a, 87824~128190ten thousand yuan/10a or 276670~399510ten thousand yuan/10a, and 36.81~52.33person/km^2^/10a, respectively. This means that these ranges probably have more contributions to local zones’ precipitation changes.

As shown in Fig. 3, the leading impact type or range of each leading factor on precipitation change is predominantly located in the northeast-southeast of the Beibu Gulf Economic Zone. This indicates that the largest precipitation change is in the northeast-southeast of the Beibu Gulf Economic Zone, and the range of the precipitation trend rate is between 39 and 96 mm/ 10a (Fig. 3). Therefore, the main distribution areas of the main impact range (type) of the leading factors on precipitation revealed by the results of the risk detector are consistent with the distribution of the relatively large area of precipitation change trend rate calculated by the linear regression model. This illustrates the flexibility of applying the geographical detector model to obtaining initial detection results of the precipitation change mechanism. Figure 3 shows that the precipitation change for the county of Rongxian is strongly controlled by FCRC, URC and PDC. According to the interaction detector, we also found that the P_D,H_values of FCRC ∩ URC (74.1%), FCRC ∩ PDC (75.0%), and URC ∩ PDC (71.0%) are very high and enhance each other to increase precipitation change, which emphasizes directions for future work. In conclusion, the largest precipitation change is present in the northeast-southeast region of the Beibu Gulf Economic Zone and is predominantly influenced by the interactions of factors such as FCRC, URC, GDPC and PDC.

## Conclusions and Discussion

The causes of precipitation changes are very complicated due to the interaction of the land surface with the atmosphere. In addition, the research resources, such as shared data, are limited in developing countries, creating a high demand for useful detecting and/or analyzing tools. In this study, we used geographical detectors to verify the effects of some of the natural and social factors on precipitation change at a regional scale. We believe that this program is unique because it extracts the interrelationships between precipitation change and terrestrial environmental factors using the correspondence of their spatial distribution and, most importantly, because it is easily implemented.

The feedback of terrestrial environment to precipitation changes can be partially explained by forest cover, urbanization, terrain, irrigation and other single factors. Typically, the comprehensive consequences are the result of interactions of multiple factors. In this study, we found the following:

1. The precipitation trend rate of the Beibu Gulf Economic Zone is between −47 mm/10a and 96 mm/10a. The minimum and maximum values occur in the southwest and northeast of the Beibu Gulf Economic Zone, respectively.
2. The results found by the factor detector and the ecological detector show that FCRC, URC, GDPC and PDC, as the leading factors of precipitation change, have a relatively large contribution to the precipitation changes.
3. The interaction of pairs of impact factors has far larger effects than the corresponding single factor does on precipitation changes.
4. The precipitation change is predominantly due to human factors, and thus, humans act as an active media linking and enhancing the other impact factors.
5. The results of the risk detector show that the main impact types (ranges) of the leading factors of FCRC, URC, GDPC and PDC on precipitation change are 0.7411~4.7979%/10a, −7.7920~2.5006%/10a, 87824~128190ten thousand yuan/10a or 276670~399510ten thousand yuan/10a, and 36.81~52.33person/km^2^/10a, respectively.

Our research suggests that the geographical detector offers a quantitative and objective analytical framework that could be used to find the essence of many geosciences phenomena. There are still several aspects for future study. First, spatial scale transformation is an important aspect of geographical detectors. Transforming the administrative regions into the same grid cells might be subjective, as the grid size can have different values. We also found that discretization methods to classify continuous variables into several categories might affect the results because these methods do not currently have standardized rules. Second, due to the limitation of range and data accessibility in this study area, quantitative analysis was not conducted overall based on impact factors in this study. Third, the main impact ranges of leading factors (FCRC, URC, GDPC and PDC) fluctuated with precipitation change, and the largest precipitation change is typically only consistent with the smallest range of URC. In the future, threshold values of the main impact ranges can be overcome by collaboration with climate models. This is likely a better way to integrate geographical detectors with traditional meteorology methods to discover the precipitation change mechanism.

Despite some limitations, we still believe that this study will be meaningful. The geographical detectors are statistical and are not a causality tool; however, they can distinguish high potential impact factors and leading factor ranges to emphasize the next step in research. The results from this study can help researchers to understand the spatial pattern of precipitation change with impact factors and provide clues for further studies by integrating traditional observation, simulation, contrast testing, etc.

## Materials and Methods

### Research methods

#### Trend rate

Tests for trend detection of the climatic element in a time series can be classified as parametric and non-parametric methods (e.g., the Mann-Kendall test). The linear regression method is a very simple and common parametric method^51^, and the trend rate method generally adopts the unitary linear regression model, that is:


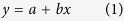


where y represents a climatic element or other sequence (e.g., precipitation); x represents a yearly time series (from 1985 to 2010); and b represents a linear trend term, the value of which is a linear trend rate, in mm/10a.

#### Geographic detector model

Geographical detectors are composed of the factor detector, ecological detector, risk detector and interaction detector^34^,^43^. Factors significantly affecting precipitation change can be selected as the leading factors through analysis using the factor detector and ecological detector models; the risk detector can further analyze leading impact types or scopes (confidence level of 95%) of impact factors that significantly affect precipitation change; and the interaction detector can analyze the interaction among various factors. The core concept of the factor detector is as follows: there is certain differentiation of the factors affecting the development of geographical phenomenon in space. If a certain factor has a remarkable consistency with the change of that geographical phenomenon in space, then the factor will have a definite determinant power on the occurrence and development of a geographical phenomenon^34^, measured by the size of the power determinant value (P_D,H_). Details of the geographical detector can be found in the original paper^34^. Here, in our research context, the calculation model for detecting impact factors of precipitation change in the Beibu Gulf Economic Zone is reviewed as follows:

We assume that precipitation change would present a spatial distribution similar to that of an impact factor if the impact factor leads to the change of precipitation (see Supplementary Fig. S2). All impact factors are quantified by these power values as follows:


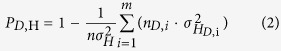


In equation (2), D represents an impact factor layer (e.g., DEM or slope) that must already be categorized (e.g., DEM values can be categorized into eight categories); m is the number of zones (categories) of the factor D (D = {D_1_,D_2_,D_3_, …, D_m_}); H represents the precipitation trend rate; 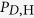 represents the power of determinant D on H; n and 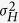 represent the number of total samples and the global variance of H over the entire study area, respectively; 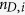 and 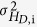 represent the number of samples in the i-th sub-regions of D (layer D) and the variance of H over the i-th sub-regions of D, respectively; and 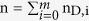. If it is a perfect division and local variance is 0 (assuming 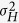
≠0), then 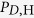
=1. In general, the value range of 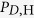
is [0, 1]^34^,^41^. P_D,H_=1 means that the impact factor stratum completely explains the spatial precipitation change, whereas P_D,H_=0 implies a completely random spatial occurrence of the precipitation change.

The ecological detector compares which suspected impact factor (e.g., C factor) determinant is more significant than the other (e.g., D factor) in causing precipitation change in the study area. This is measured using the F-test:


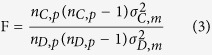


In equation (3), F is the test value of F, n_C,p_ and n_D_,_p_ denote the number of samples of impact factors C and D in sample unit p, respectively, and 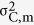 and 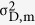 are dispersion variances of impact factors C and D, respectively. The null hypothesis is H_0_ : 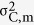 = 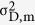. If H_0_ is rejected conditioned on a significant level α (usually 5%), we conclude that the impact factor C is more significant than the impact factor D in affecting precipitation change.

Different types or ranges of an impact factor have different influences on precipitation change. The risk detector compares the differences through the t-test. The computational formula is as follows:


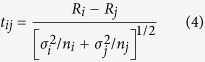


In equation (4), t_ij_ is the test value of t; R_i_ and R_j_ are average values of the precipitation tendency rate over property i and property j of the impact factor R; 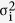 and 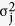 are the variances of the precipitation tendency rate from property i and property j, respectively; and 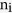 and 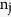 are the sample sizes of the two properties.

The interaction detector shows that when the two different factors of x and y are combined, they either weaken or enhance each another or they are independent in changing precipitation, determined by comparing P_D,H_(x ∩ y) with the values of P_D,H_(x) and P_D,H_(y), where the symbol ‘ ∩ ’ denotes the intersection between the x layer and y layer. If P_D,H_(x ∩ y) < min (P_D,H_(x), P_D,H_(y)), the variables nonlinearly weaken each other; if min (P_D,H_(x), P_D,H_(y)) < P_D,H_(x ∩ y) < max (P_D,H_(x), P_D,H_(y)), the variables uniweaken each other; if P_D,H_(x ∩ y) > max (P_D,H_(x), P_D,H_(y)), the variables bienhance each other; and if P_D,H_(x ∩ y) > P_D,H_(x) + P_D,H_(y), the variables nonlinearly enhance each other. If P_D,H_(x ∩ y) = P_D,H_(x) + P_D,H_(y), then the variables are independent of each other.

Based on the precipitation trend rate in the Beibu Gulf Economic Zone from 1985 to 2010, geographical detectors were utilized to explore the impact and indication effect of terrestrial environmental factors on the precipitation change through climate feedbacks.

#### Technical process

Modeling of the geographical detector mainly involves the following steps: first, determination of the optimal classification method for the factor data; second, determination of the impact of factors on the precipitation change; and third, determination of the leading role of factors in the precipitation change. Regarding the technical process in detail, please see Supplementary Fig. S3.

#### Data sources and processing

##### Precipitation data

**Figure 1:**
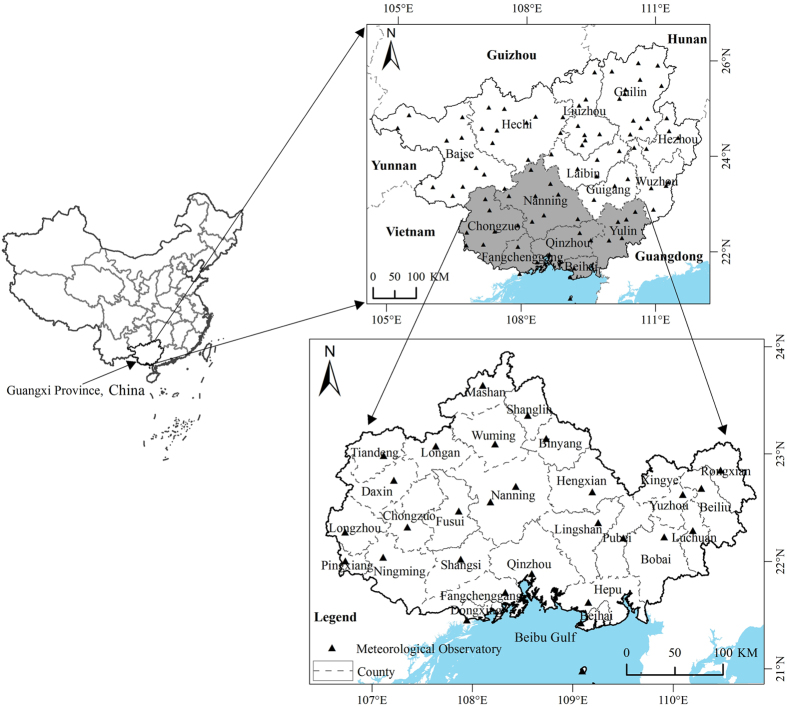
Location map for the study area showing the 29 meteorological observatories selected from 88 meteorological observatories located in Guangxi Province. It was generated by ArcGIS10.1(http://www.esrichina.com.cn/softwareproduct/ArcGIS/); the locations of the meteorological observatories were obtained from the China meteorological data network (http://data.cma.cn).

**Figure 2:**
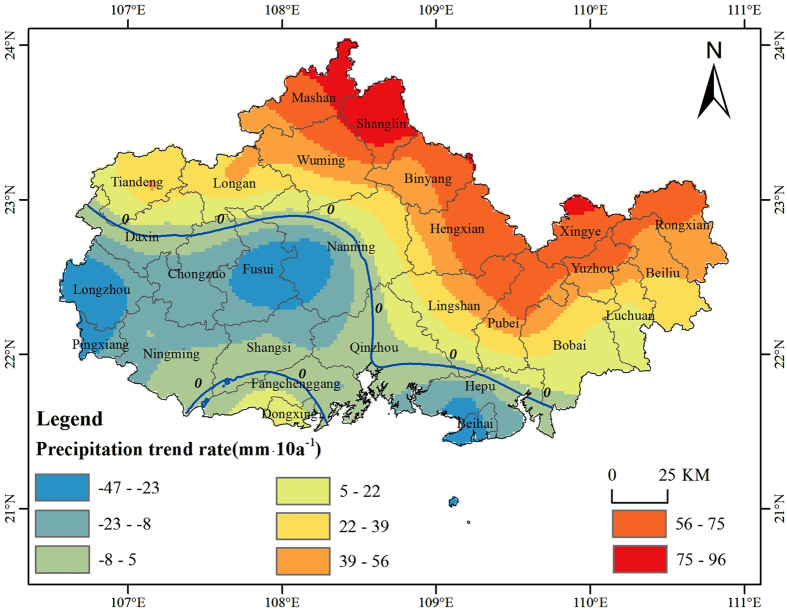
The spatial distribution of precipitation changes in the study area. It was generated using ArcGIS 10.1 (http://www.esrichina.com.cn/softwareproduct/ArcGIS/).

**Figure 3:**
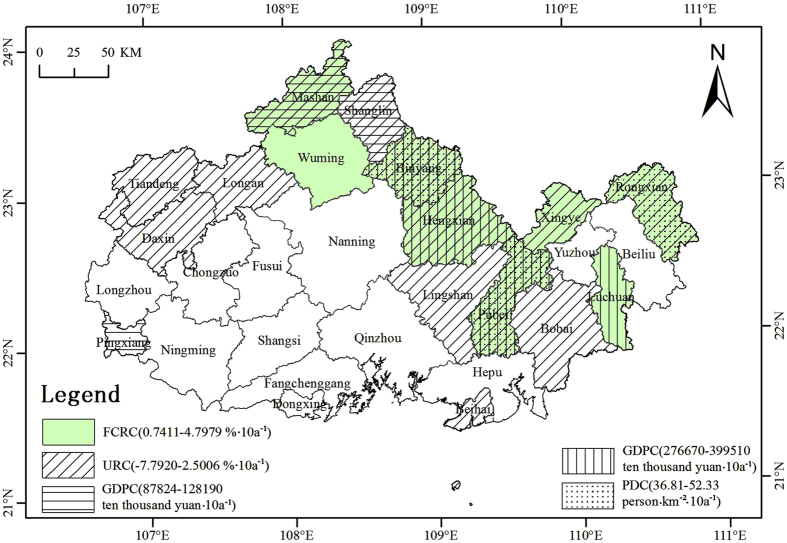
Distribution map of the leading impact type or range of each leading factor on precipitation change in the study area. The map was generated using ArcGIS 10.1 (http://www.esrichina.com.cn/softwareproduct/ArcGIS/).

The selected observation data are the mean annual precipitation of 29 meteorological stations from 1985 to 2010 in the Beibu Gulf Economic Zone (Fig. 1). The above data were derived from the China meteorological data network (http://data.cma.cn).

##### Potential natural factors

According to the main impact factors of precipitation change discussed in the introduction, almost all of the environmental factors, except for climate type, were considered as main potential natural factors, such as geomorphic type, the types of terrestrial ecosystem, vegetation type, elevation, gradient, aspect and forest coverage rate, to reveal the feedback. Based on the results of China’s ecological geographic division, the entire study area belongs to the climate type of the south subtropical-humid region; thus, the climate type factor can be ruled out here.

During the study period (1985–2010), the elevation, gradient and aspect of the Guangxi Beibu Gulf Economic Zone remain relatively stable, so SRTM DEM was used and also to produce the gradient and aspect. Similarly, the changes of geomorphic type, the type of terrestrial ecosystems and vegetation type were relatively small and fragmented, so we selected a middle year (around 2000) of these datasets to represent the entire study period. The datasets above were provided by the Data Center for Resources and Environmental Sciences, Chinese Academy of Sciences (RESDC) (http://www.resdc.cn). Annual forest coverage rates were collected from the Guangxi Forestry Yearbooks (1958–2003) and the Guangxi Statistical Yearbooks. To ensure the continuity of the dataset in time, regression analysis was used to fix missing data.

**Figure 4:**
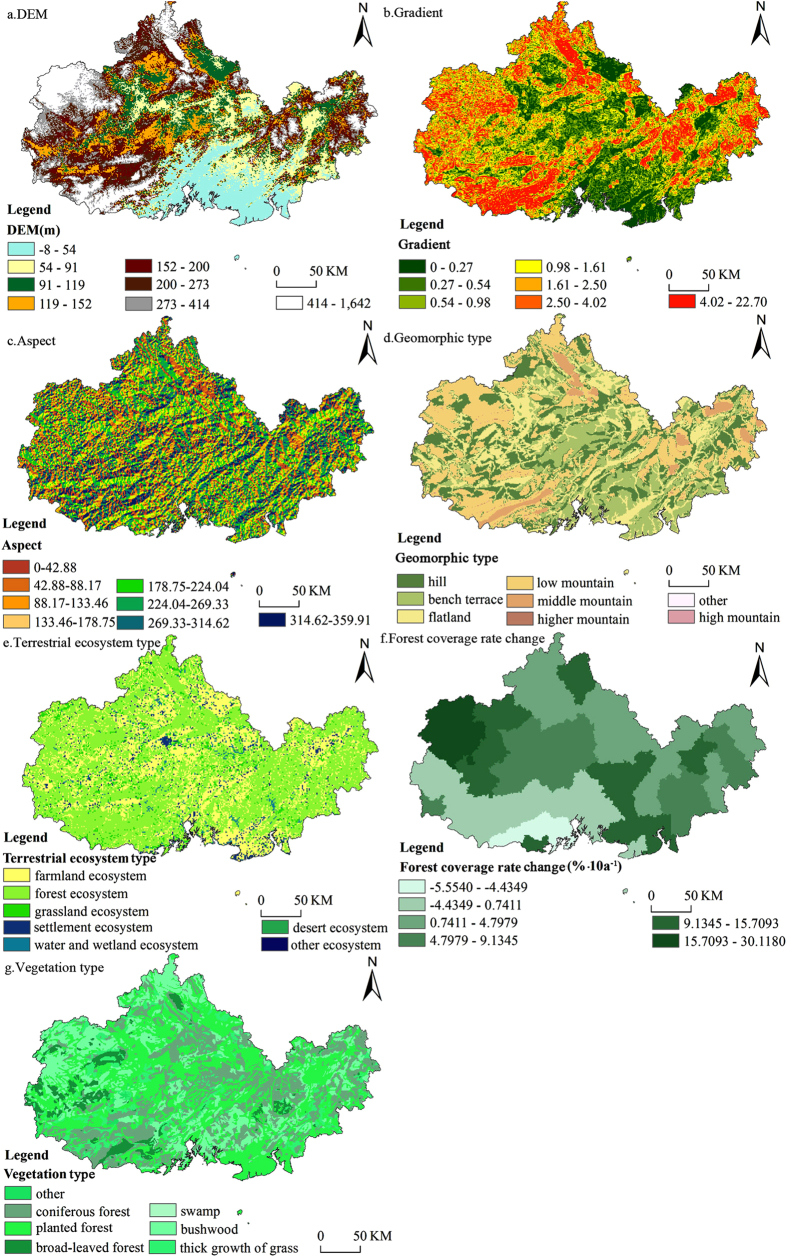
Spatial distribution of potential natural factors in the study area. This map was generated by ArcGIS 10.1 (http://www.esrichina.com.cn/softwareproduct/ArcGIS/). (**a**) DEM with the sea-level elevation data at the resolution of 90 m of SRTM, which was divided into 8 types through QV; (**b**) Gradient data results obtained from the gradient analysis on DEM in ArcGIS, which was divided into 7 types through QV; (**c**) Aspect map results from the analysis of aspect on DEM in ArcGIS, which was divided into 8 types through NB; (**d**) Geomorphic type derived from a 1: 1,000,000 geomorphic map at the spatial resolution of 1000 * 1000 m; (**e**) Data of the terrestrial ecosystem type were derived from spatial distribution data of the Chinese terrestrial ecosystem types at the spatial resolution of 1000 * 1000 m; (**f**) Forest coverage rate change was calculated through Equation (1), and then, the trend of the forest coverage rate of each county (b value) was mapped and classified into 6 types through NB; (**g**) Data of the vegetation types were derived from a 1: 1,000,000 vegetation map at the spatial resolution of 1000 * 1000 m.

Data sorting and pretreatment were conducted in ArcGIS10.1. Based on the input requirements of the geographical detector model (http://www.sssampling.org/Excel-GeoDetector/), projection was unified to the projection coordinate system of Krasovsky-1940-Albers, and raster data were reclassified as 6 to 8 grades^27^,^36^,^37^ and then converted to the vector data type. ArcGIS provided some discrete classification methods, such as the Equal Interval Method (EI), Quantile Value Method (QV), Natural Break Method (NB) and Geometrical Interval Method (GI), to reclassify the raster data. Different classification methods result in different P_D,H_ values for the classified factor. The highest P_D,H_ value result will indicate that this impact factor classification, using the discrete method, can be more representative as the classification of a geographical phenomenon, thus better revealing spatial distribution laws of the geographical phenomenon^38^. Natural factors were processed and classification methods were selected after many experiments (Fig. 4).

##### Potential social factors

**Figure 5:**
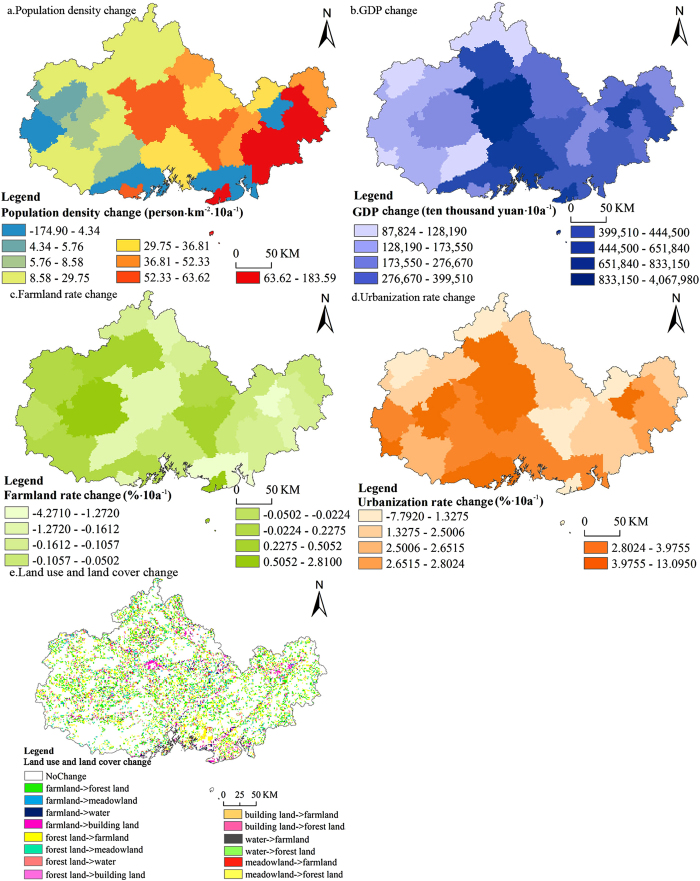
Spatial distribution map of the potential social factors in the study area. This map was generated by ArcGIS 10.1. (http://www.esrichina.com.cn/softwareproduct/ArcGIS/). (**a**) Population density change was calculated using Equation (1), and then, the trend of the annual population density of each county from 1985 to 2010 (b value) was mapped and classified into 8 types through QV; (**b**) GDP change was calculated using Equation (1), and then, the trend of the annual GDP of each county from 1985 to 2010 (b value) was mapped and divided into 8 types through QV; (**c**) Farmland rate change was calculated using Equation (1), and then, the trend of the annual farmland rate of each county from 1985 to 2010 (b value) was mapped and divided into 8 types through QV; (**d**) Urbanization rate change was calculated using Equation (1), and then, the trend of the annual urbanization rate of each county from 1985 to 2010 (b value) was mapped and divided into 6 types through GI; (**e**) Landuse and landcover change was derived from the subtraction of land use maps between the 1980 s and 2010.

Population density, GDP, farmland rate, urbanization rate and land use were selected as potential social factors that likely caused regional precipitation change because of changes in them, as described in the introduction. The population density, GDP and urbanization rate were derived from the Guangxi Statistical Yearbooks (1986~1991, 1993~1999 and 2001~2010), while the farmland rate was derived from the Guangxi Rural Statistical Yearbooks (1985~2010), and regression analysis methods were used to fill the entire 26-year period (1985–2010). Land use data (1980s and 2010) were collected from the Data Center for Resources and Environmental Sciences, Chinese Academy of Sciences (RESDC) (http://www.resdc.cn). We used the trend rates of these factors, derived from Equation (1), to express change because these factors have changed greatly over the 26-yearperiod. Similarly, the social factors adopted the same processing method as the potential natural factors (Fig. 5).

## Additional Information

**How to cite this article**: Zhao, Y. *et al*. Quantitative analysis of the impacts of terrestrial environmental factors on precipitation variation over the Beibu Gulf Economic Zone in Coastal Southwest China. *Sci. Rep.* **7**, 44412; doi: 10.1038/srep44412 (2017).

**Publisher's note:** Springer Nature remains neutral with regard to jurisdictional claims in published maps and institutional affiliations.

## Supplementary Material

Supplementary Information

## Figures and Tables

**Table 1:** Sorting of the terrestrial environmental factors and their P_D,H_ values relating to precipitation change.

Index	Terrestrial environmental factors	Factor codes	P_D,H_ values	Remarks
Precipitation trend rate	Forest coverage rate change	FCRC	50.3%	Natural impact
	Urbanization rate change	URC	47.3%	Social impact
	GDP change	GDPC	43.5%	Social impact
	Farmland rate change	FRC	35.2%	Social impact
	Population density change	PDC	27.4%	Social impact
	Vegetation type	VT	10.0%	Natural impact
	DEM	DEM	7.3%	Natural impact
	Geomorphic type	GT	2.4%	Natural impact
	Gradient	GRD	0.8%	Natural impact
	Aspect	ASP	0.4%	Natural impact
	Land use and land cover change	LUCC	0.3%	Social impact
	Terrestrial ecosystem type	TET	0.1%	Natural impact

**Table 2:** Statistically significant difference of influence factors on precipitation change.

Difference	FCRC	URC	GDPC	FRC	PDC	VT	DEM	GT	GRD	ASP	LUCC	TET
**FCRC**												
**URC**	N											
**GDPC**	N	N										
**FRC**	N	Y	Y									
**PDC**	N	N	N	N								
VT	N	N	N	N	N							
DEM	N	N	N	N	N	Y						
GT	N	N	N	N	N	Y	N					
GRD	N	N	N	N	N	Y	N	N				
ASP	N	N	N	N	N	Y	N	N	N			
LUCC	N	N	N	N	N	N	N	N	N	N		
TET	N	N	N	N	N	Y	N	N	N	N	N	

Y means the difference of the influence between the two factors is significant with a confidence of 95%, while N means no significant difference.

**Table 3:** Effects of interactions (measured by the P_D,H_ value)between pairs of factors on precipitation changes.

Interaction	FCRC	URC	GDPC	FRC	PDC	VT	DEM	GT	GRD	ASP	LUCC	TET
**FCRC**												
**URC**	B											
**GDPC**	B	B										
FRC	B	B	A									
**PDC**	B	B	B	A								
VT	B	B	A	A	A							
DEM	B	A	A	A	A	A						
GT	A	A	A	A	A	A	A					
GRD	A	A	A	A	A	A	A	B				
ASP	A	A	A	A	A	A	A	A	A			
LUCC	A	A	A	A	A	A	A	A	A	A		
TET	A	A	A	A	A	A	A	A	A	A	A	

A means nonlinear enhancement and B means bienhancement.

**Table 4:** Main impact ranges of social factors on precipitation change in the study area.

Index	Leading factors	Impact type (range) of leading factors		Mean value of Precipitation change
		Grade of Types	Values	
Precipitation trend rate (mm/10a)	FCRC (%/10a)	I	0.7411~4.7979	53.18
	URC (%/10a)	I~II	−7.7920~2.5006	41.42~41.54
	GDPC (ten thousand yuan/10a)	I	87824~128190	50.33
		IV	276670~399510	49.34
	PDC (person/km^2^/10a)	VI	36.81~52.33	41.97
