# Supplementary material for: Quantitative analysis of the impacts of terrestrial environmental factors on precipitation variation over the Beibu Gulf Economic Zone in Coastal Southwest China
Source: Sci Rep. 2017 Mar 15;7:44412. doi: 10.1038/srep44412 (PMC5353577; doi:10.1038/srep44412)
Supplement: Supplementary Information [file srep44412-s1.pdf]

Quantitative analysis of the impacts of terrestrial environmental factors on precipitation variation over the Beibu Gulf Economic Zone in Coastal Southwest China

Yinjun Zhao[1,*],Qiyu Deng[2],Qing Lin[1] & Chunting Cai[2]

[1] Key Laboratory of Environment Change and Resources Use in Beibu Gulf, Ministry of Education,

Guangxi Teachers Education University, 175 Mingxiu east st，Nanning 530001,China;crpp0104@163.com

[2] School of Geography and Planning, Guangxi Teachers Education University, 175 Mingxiu east st，

Nanning 530001,China;

Table S1 Annual precipitation trends of meteorological observatories with statistical significance

Station ID	Precipitation trend rate(mm/10a)	Station ID	Precipitation trend rate (mm/10a)	Station ID	Precipitation trend rate (mm/10a)
59246**	132.08	59254	91.478	59012	-25.188
59034**	144	59227#	71.653	59644#	-41.257
59055*	121.43	59041	65.673	59001	-15.945
59038*	111.93	59431#	-68.545	59626#	47.01
59046*	139.04	59249	71.654	59051	20.276
59235#	120.79	59237#	51.195	59015	25.909
59058	123.2	59230#	57.603	59451#	24.082
59241	104.44	59265	58.512	59215	19.579
59229#	76.44	57954	80.692	59017	15.013
59045	109.07	59025	-74.033	59218	-17.115
59037	118.2	57947	64.089	59044	16.602
59064	108.16	57957	74.272	57941	-17.389
57948	107.71	59448#	89.266	59224	13.582
59047	116.02	59452#	64.341	59023	-15.944
59441#	86.96	59446#	66.182	59027	13.555
57964	76.735	59209	-37.242	57942	11.176
59211	78.97	57927	-36.226	59631#	-17.958
57956	115.34	59454	49.735	59449#	11.719
57859	-93.684	59256	45.508	59632#	10.034
59065	107.1	59052	46.244	59004	-3.761
59255	118.99	59033	51.904	59021	6.25
59453#	96.951	59213	33.326	59228	2.966
59053	78.361	59419#	-31.158	59457#	7.718
59059	111.94	59022	-31.954	59031	3.871
59242	78.155	59238#	26.708	59427#	1.814
57955	87.56	59640#	-51.814	59421#	2.187
59266	62.243	59425	-27.901	59435#	-2.235
59426#	-74.266	59061	34.148	59429#	0.365
59417#	-69.518	59647#	-35.277		
59057	76.064	57960	23.563		

** and * Asterisk indicate values siginificant at the 0.05 level and 0.01 level resepectively. # marks indicate these stations locates at the Guangxi Beibu Gulf Economic Zone.

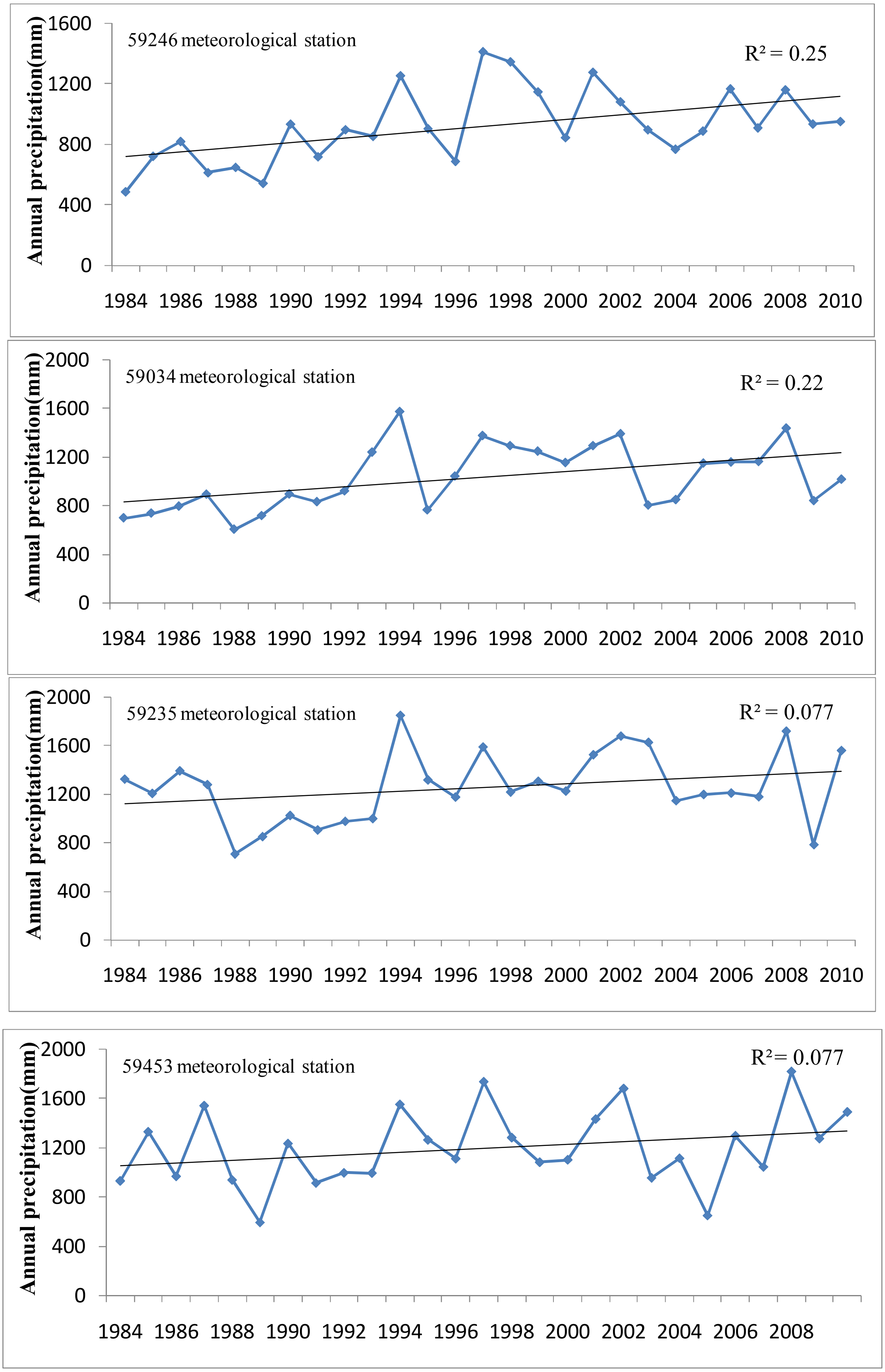

Figure S1. Annual precipitation treads of 4 meteorological stations.

Table S2 **Sorted interactions (measured by $P_{D,H}$ value) between pairs of impact factors**

C	A+B	Results	Interpretation
FRC∩GDPC=0.844	>0.787=FRC(0.352)+GDPC(0.435)	C>A+B	Nonlinear enhancement
FCRC∩GDPC=0.831	<0.938=FCRC(0.503)+GDPC(0.435)	C>A,B;C<A+B	Bi-enhancement
FRC∩URC=0.77	<0.825=FRC(0.352)+URC(0.473)	C>A,B;C<A+B	Bi-enhancement
FCRC∩FRC=0.765	<0.855=FCRC(0.503)+FRC(0.352)	C>A,B;C<A+B	Bi-enhancement
FCRC∩PDC=0.75	<0.777=FCRC(0.503)+PDC(0.274)	C>A,B;C<A+B	Bi-enhancement
FCRC∩URC=0.741	<0.976=FCRC(0.503)+URC(0.473)	C>A,B;C<A+B	Bi-enhancement
URC∩GDPC=0.723	<0.908=URC(0.473)+GDPC(0.435)	C>A,B;C<A+B	Bi-enhancement
URC∩PDC=0.71	<0.747=URC(0.473)+PDC(0.274)	C>A,B;C<A+B	Bi-enhancement
FRC∩PDC=0.701	>0.626=FRC(0.352)+PDC(0.274)	C>A+B	Nonlinear enhancement
GDPC∩PDC=0.7	<0.709=GDPC(0.435)+PDC(0.274)	C>A,B;C<A+B	Bi-enhancement
FCRC∩VT=0.598	<0.603=FCRC(0.503)+VT(0.100)	C>A,B;C<A+B	Bi-enhancement
FCRC∩DEM=0.573	<0.576=FCRC(0.503)+DEM(0.073)	C>A,B;C<A+B	Bi-enhancement
GDPC∩DEM=0.57	>0.508=GDPC(0.435)+DEM(0.073)	C>A+B	Nonlinear enhancement
GDPC∩VT=0.569	>0.536=GDPC(0.435)+VT(0.100)	C>A+B	Nonlinear enhancement
URC∩DEM=0.552	>0.545=URC(0.473)+DEM(0.073)	C>A+B	Nonlinear enhancement
URC∩VT=0.55	<0.573=URC(0.473)+VT(0.100)	C>A,B;C<A+B	Bi-enhancement
FRC∩DEM=0.549	>0.424=FRC(0.352)+DEM(0.073)	C>A+B	Nonlinear enhancement
FCRC∩GT=0.548	>0.527=FCRC(0.503)+GT(0.024)	C>A+B	Nonlinear enhancement
FCRC∩GRD=0.536	>0.511=FCRC(0.503)+GRD(0.008)	C>A+B	Nonlinear enhancement
FCRC∩TET=0.51	>0.504=FCRC(0.503)+TET(0.0009)	C>A+B	Nonlinear enhancement
FCRC∩LUCC=0.51	>0.506=FCRC(0.503)+LUCC(0.003)	C>A+B	Nonlinear enhancement
FCRC∩ASP=0.509	>0.507=FCRC(0.503)+ASP(0.004)	C>A+B	Nonlinear enhancement
URC∩GRD=0.5	>0.481=URC(0.473)+GRD(0.008)	C>A+B	Nonlinear enhancement
URC∩GT=0.5	>0.497=URC(0.473)+GT(0.024)	C>A+B	Nonlinear enhancement
GDPC∩GT=0.5	>0.459=GDPC(0.435)+GT(0.024)	C>A+B	Nonlinear enhancement
URC∩TET=0.485	>0.474=URC(0.473)+TET(0.0009)	C>A+B	Nonlinear enhancement
GDPC∩GRD=0.483	>0.443=GDPC(0.435)+GRD(0.008)	C>A+B	Nonlinear enhancement
URC∩ASP=0.48	>0.476=URC(0.473)+ASP(0.004)	C>A+B	Nonlinear enhancement
URC∩LUCC=0.48	>0.476=URC(0.473)+LUCC(0.003)	C>A+B	Nonlinear enhancement
FRC∩VT=0.474	>0.452=FRC(0.352)+VT(0.100)	C>A+B	Nonlinear enhancement
GDPC∩TET=0.455	>0.436=GDPC(0.435)+TET(0.0009)	C>A+B	Nonlinear enhancement
GDPC∩LUCC=0.447	>0.438=GDPC(0.435)+LUCC(0.003)	C>A+B	Nonlinear enhancement
GDPC∩ASP=0.443	>0.439=GDPC(0.435)+ASP(0.004)	C>A+B	Nonlinear enhancement
PDC∩VT=0.433	>0.374=PDC(0.274)+VT(0.100)	C>A+B	Nonlinear enhancement
FRC∩GRD=0.411	>0.36=FRC(0.352)+GRD(0.008)	C>A+B	Nonlinear enhancement
PDC∩DEM=0.403	>0.347=PDC(0.274)+DEM(0.073)	C>A+B	Nonlinear enhancement
FRC∩GT=0.402	>0.376=FRC(0.352)+GT(0.024)	C>A+B	Nonlinear enhancement
FRC∩TET=0.37	>0.353=FRC(0.352)+TET(0.0009)	C>A+B	Nonlinear enhancement

FRC∩LUCC=0.365	>0.355=FRC(0.352)+LUCC(0.003)	C>A+B	Nonlinear enhancement
FRC∩ASP=0.361	>0.356=FRC(0.352)+ASP(0.004)	C>A+B	Nonlinear enhancement
PDC∩GT=0.314	>0.298=PDC(0.274)+GT(0.024)	C>A+B	Nonlinear enhancement
PDC∩GRD=0.3	>0.282=PDC(0.274)+GRD(0.008)	C>A+B	Nonlinear enhancement
PDC∩ASP=0.286	>0.278=PDC(0.274)+ASP(0.004)	C>A+B	Nonlinear enhancement
PDC∩LUCC=0.283	>0.277=PDC(0.274)+LUCC(0.003)	C>A+B	Nonlinear enhancement
PDC∩TET=0.281	>0.275=PDC(0.274)+TET(0.0009)	C>A+B	Nonlinear enhancement
DEM∩VT=0.191	>0.173=DEM(0.073)+VT(0.100)	C>A+B	Nonlinear enhancement
GT∩VT=0.14	>0.124=GT(0.024)+VT(0.100)	C>A+B	Nonlinear enhancement
GRD∩VT=0.131	>0.109=GRD(0.008)+VT(0.100)	C>A+B	Nonlinear enhancement
DEM∩GT=0.118	>0.097=DEM(0.073)+GT(0.024)	C>A+B	Nonlinear enhancement
VT∩LUCC=0.111	>0.103=VT(0.100)+LUCC(0.003)	C>A+B	Nonlinear enhancement
ASP∩VT=0.11	>0.104=ASP(0.004)+VT(0.100)	C>A+B	Nonlinear enhancement
TET∩VT=0.106	>0.101=TET(0.0009)+VT(0.100)	C>A+B	Nonlinear enhancement
DEM∩GRD=0.095	>0.081=DEM(0.073)+GRD(0.008)	C>A+B	Nonlinear enhancement
DEM∩LUCC=0.085	>0.076=DEM(0.073)+LUCC(0.003)	C>A+B	Nonlinear enhancement
DEM∩TET=0.082	>0.074=DEM(0.073)+TET(0.0009)	C>A+B	Nonlinear enhancement
DEM∩ASP=0.081	>0.076=DEM(0.073)+ASP(0.004)	C>A+B	Nonlinear enhancement
GT∩LUCC=0.035	>0.027=GT(0.024)+LUCC(0.003)	C>A+B	Nonlinear enhancement
ASP∩GT=0.032	>0.028=ASP(0.004)+GT(0.024)	C>A+B	Nonlinear enhancement
GRD∩GT=0.03	<0.032=GRD(0.008)+GT(0.024)	C>A,B;C<A+B	Bi-enhancement
TET∩GT=0.03	>0.025=TET(0.0009)+GT(0.024)	C>A+B	Nonlinear enhancement
GRD∩LUCC=0.022	>0.011=GRD(0.008)+LUCC(0.003)	C>A+B	Nonlinear enhancement
GRD∩ASP=0.02	>0.012=GRD(0.008)+ASP(0.004)	C>A+B	Nonlinear enhancement
ASP∩LUCC=0.014	>0.007=ASP(0.004)+LUCC(0.003)	C>A+B	Nonlinear enhancement
GRD∩TET=0.013	>0.009=GRD(0.008)+TET(0.0009)	C>A+B	Nonlinear enhancement
TET∩LUCC=0.009	>0.004=TET(0.0009)+LUCC(0.003)	C>A+B	Nonlinear enhancement
ASP∩TET=0.008	>0.005=ASP(0.004)+TET(0.0009)	C>A+B	Nonlinear enhancement

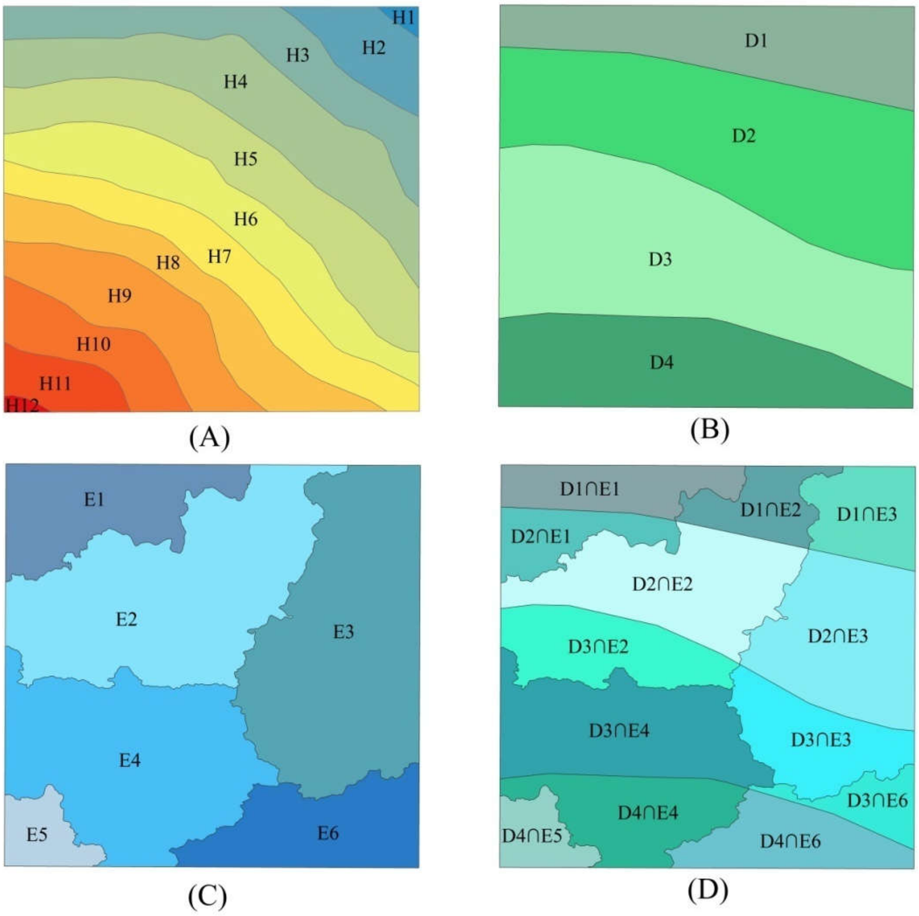

Figure S2. Diagram illustrating geographical detector method. **(A)** Spatial distrbution of trend rates of annual pericipitation changes which effected by factor D , factor E and factor D∩E at spatial-temporal scale; **(B)** Factor layer D including four categories (D1, D2, D3 and D4); **(C)** Factor layer E including six categories (E1, E2, E3, E4, E5 and E6); **(D)** Interaction of factors D and E (D∩E) that can be done through overlaying in ArcGIS. These maps were generated using ArcGIS10.1(http://www.esrichina.com.cn/softwareproduct/ArcGIS/)

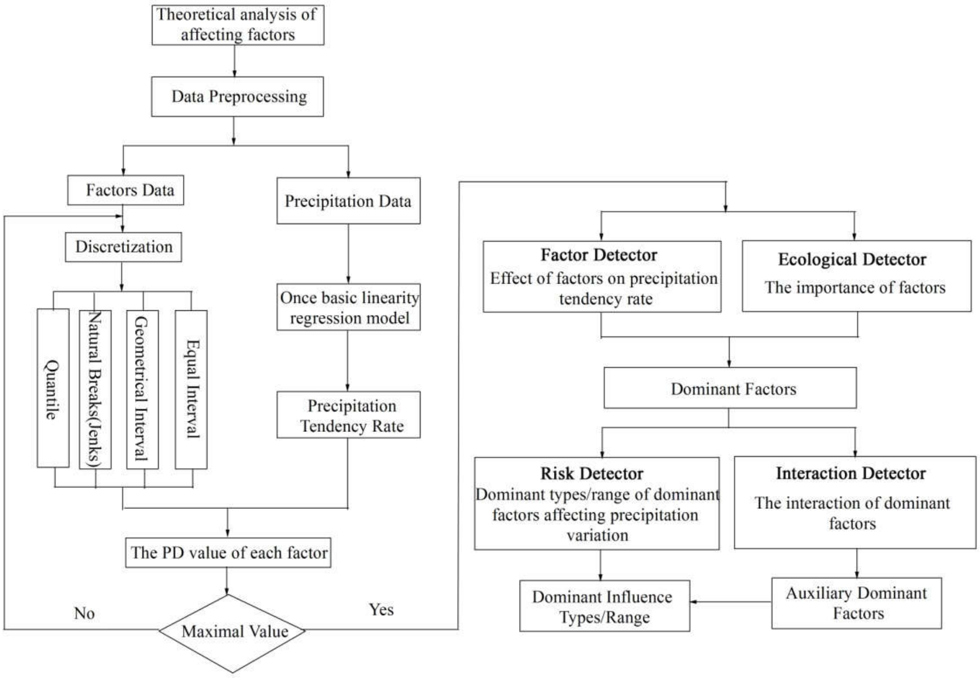

Figure S3. Technical flowchart
